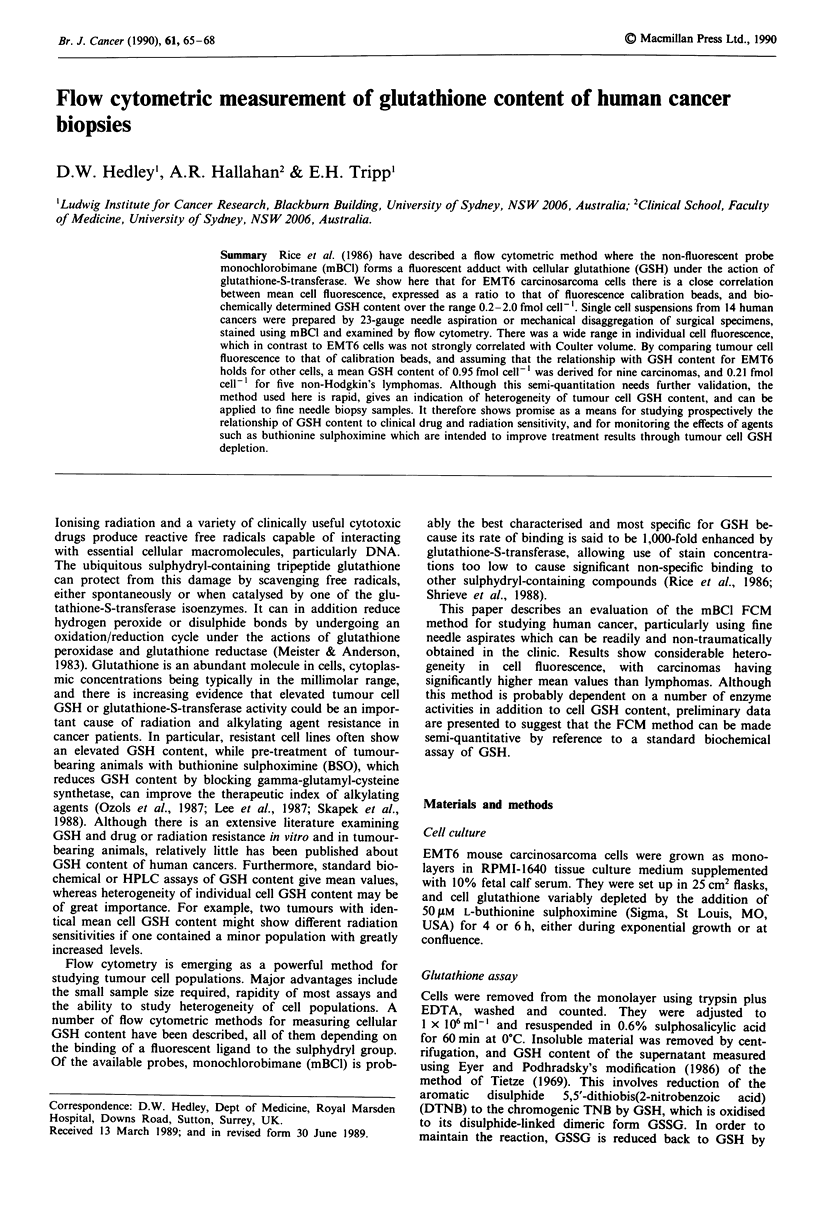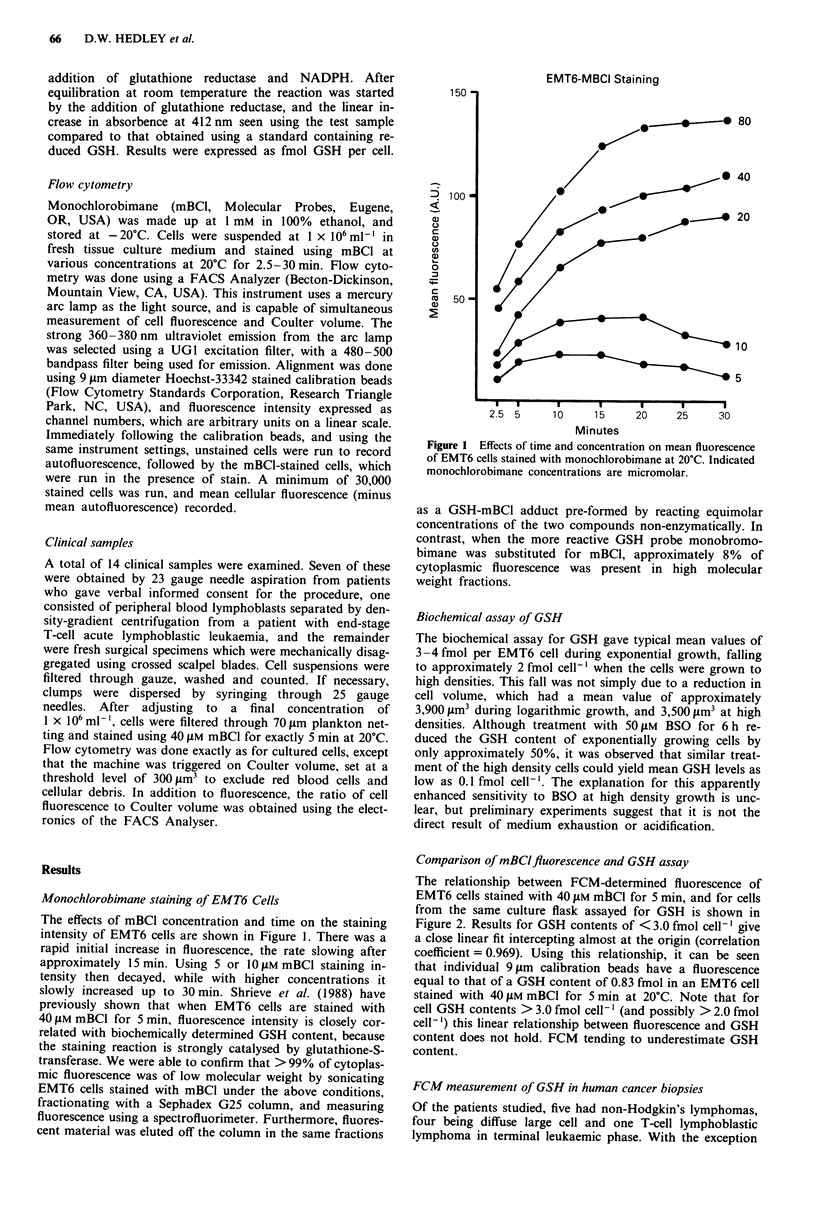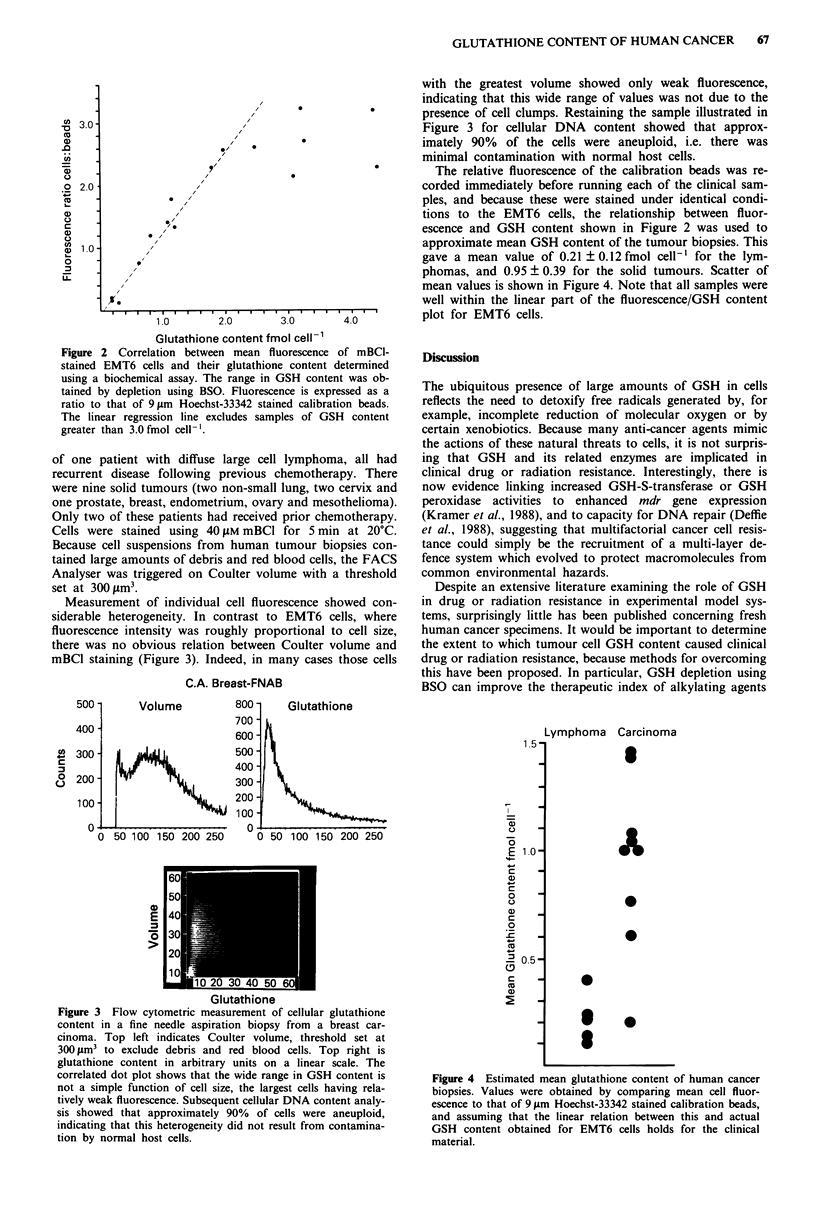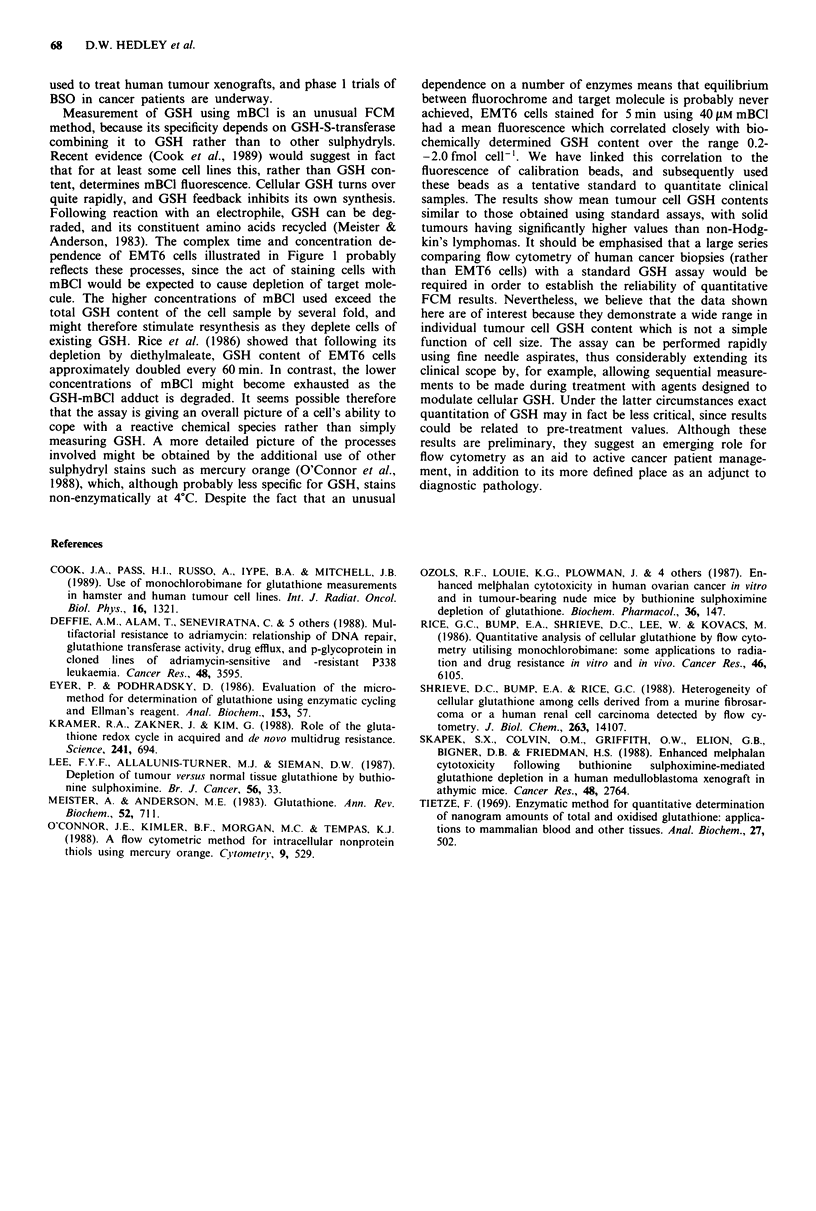# Flow cytometric measurement of glutathione content of human cancer biopsies.

**DOI:** 10.1038/bjc.1990.14

**Published:** 1990-01

**Authors:** D. W. Hedley, A. R. Hallahan, E. H. Tripp

**Affiliations:** Ludwig Institute for Cancer Research, University of Sydney, NSW, Australia.

## Abstract

Rice et al. (1986) have described a flow cytometric method where the non-fluorescent probe monochlorobimane (mBCl) forms a fluorescent adduct with cellular glutathione (GSH) under the action of glutathione-S-transferase. We show here that for EMT6 carcinosarcoma cells there is a close correlation between mean cell fluorescence, expressed as a ratio to that of fluorescence calibration beads, and biochemically determined GSH content over the range 0.2-2.0 fmol cell-1. Single cell suspensions from 14 human cancers were prepared by 23-gauge needle aspiration or mechanical disaggregation of surgical specimens, stained using mBCl and examined by flow cytometry. There was a wide range in individual cell fluorescence, which in contrast to EMT6 cells was not strongly correlated with Coulter volume. By comparing tumour cell fluorescence to that of calibration beads, and assuming that the relationship with GSH content for EMT6 holds for other cells, a mean GSH content of 0.95 fmol cell-1 was derived for nine carcinomas, and 0.21 fmol cell-1 for five non-Hodgkin's lymphomas. Although this semi-quantitation needs further validation, the method used here is rapid, gives an indication of heterogeneity of tumour cell GSH content, and can be applied to fine needle biopsy samples. It therefore shows promise as a means for studying prospectively the relationship of GSH content to clinical drug and radiation sensitivity, and for monitoring the effects of agents such as buthionine sulphoximine which are intended to improve treatment results through tumour cell GSH depletion.


 © Macmillan Press Ltd., 1990

### Flow cytometric measurement of glutathione content of human cancer biopsies

D.W. Hedley[1], A.R. Hallahan[2] & E.H. Tripp[1]

[1]*Ludwig Institute for Cancer Research, Blackburn Building, University of Sydney, NSW 2006, Australia;* [2]*Clinical School, Faculty of Medicine, University of Sydney, NSW 2006, Australia.*

**Summary** Rice *et al.* (1986) have described a flow cytometric method where the non-fluorescent probe monochlorobimane (mBCl) forms a fluorescent adduct with cellular glutathione (GSH) under the action of glutathione-S-transferase. We show here that for EMT6 carcinosarcoma cells there is a close correlation between mean cell fluorescence, expressed as a ratio to that of fluorescence calibration beads, and biochemically determined GSH content over the range 0.2–2.0 fmol $cell^{-1}$. Single cell suspensions from 14 human cancers were prepared by 23-gauge needle aspiration or mechanical disaggregation of surgical specimens, stained using mBCl and examined by flow cytometry. There was a wide range in individual cell fluorescence, which in contrast to EMT6 cells was not strongly correlated with Coulter volume. By comparing tumour cell fluorescence to that of calibration beads, and assuming that the relationship with GSH content for EMT6 holds for other cells, a mean GSH content of 0.95 fmol $cell^{-1}$ was derived for nine carcinomas, and 0.21 fmol $cell^{-1}$ for five non-Hodgkin's lymphomas. Although this semi-quantitation needs further validation, the method used here is rapid, gives an indication of heterogeneity of tumour cell GSH content, and can be applied to fine needle biopsy samples. It therefore shows promise as a means for studying prospectively the relationship of GSH content to clinical drug and radiation sensitivity, and for monitoring the effects of agents such as buthionine sulphoximine which are intended to improve treatment results through tumour cell GSH depletion.

Ionising radiation and a variety of clinically useful cytotoxic drugs produce reactive free radicals capable of interacting with essential cellular macromolecules, particularly DNA. The ubiquitous sulphydryl-containing tripeptide glutathione can protect from this damage by scavenging free radicals, either spontaneously or when catalysed by one of the glutathione-S-transferase isoenzymes. It can in addition reduce hydrogen peroxide or disulphide bonds by undergoing an oxidation/reduction cycle under the actions of glutathione peroxidase and glutathione reductase (Meister & Anderson, 1983). Glutathione is an abundant molecule in cells, cytoplasmic concentrations being typically in the millimolar range, and there is increasing evidence that elevated tumour cell GSH or glutathione-S-transferase activity could be an important cause of radiation and alkylating agent resistance in cancer patients. In particular, resistant cell lines often show an elevated GSH content, while pre-treatment of tumour-bearing animals with buthionine sulphoximine (BSO), which reduces GSH content by blocking gamma-glutamyl-cysteine synthetase, can improve the therapeutic index of alkylating agents (Ozols *et al.*, 1987; Lee *et al.*, 1987; Skapek *et al.*, 1988). Although there is an extensive literature examining GSH and drug or radiation resistance *in vitro* and in tumour-bearing animals, relatively little has been published about GSH content of human cancers. Furthermore, standard biochemical or HPLC assays of GSH content give mean values, whereas heterogeneity of individual cell GSH content may be of great importance. For example, two tumours with identical mean cell GSH content might show different radiation sensitivities if one contained a minor population with greatly increased levels.

Flow cytometry is emerging as a powerful method for studying tumour cell populations. Major advantages include the small sample size required, rapidity of most assays and the ability to study heterogeneity of cell populations. A number of flow cytometric methods for measuring cellular GSH content have been described, all of them depending on the binding of a fluorescent ligand to the sulphydryl group. Of the available probes, monochlorobimane (mBCl) is probably the best characterised and most specific for GSH because its rate of binding is said to be 1,000-fold enhanced by glutathione-S-transferase, allowing use of stain concentrations too low to cause significant non-specific binding to other sulphydryl-containing compounds (Rice *et al.*, 1986; Shrieve *et al.*, 1988).

This paper describes an evaluation of the mBCl FCM method for studying human cancer, particularly using fine needle aspirates which can be readily and non-traumatically obtained in the clinic. Results show considerable heterogeneity in cell fluorescence, with carcinomas having significantly higher mean values than lymphomas. Although this method is probably dependent on a number of enzyme activities in addition to cell GSH content, preliminary data are presented to suggest that the FCM method can be made semi-quantitative by reference to a standard biochemical assay of GSH.

### Materials and methods

### Cell culture

EMT6 mouse carcinosarcoma cells were grown as monolayers in RPMI-1640 tissue culture medium supplemented with 10% fetal calf serum. They were set up in 25 $cm^2$ flasks, and cell glutathione variably depleted by the addition of 50 μM L-buthionine sulphoximine (Sigma, St Louis, MO, USA) for 4 or 6 h, either during exponential growth or at confluence.

### Glutathione assay

Cells were removed from the monolayer using trypsin plus EDTA, washed and counted. They were adjusted to $1 \times 10^6\,ml^{-1}$ and resuspended in 0.6% sulphosalicylic acid for 60 min at 0°C. Insoluble material was removed by centrifugation, and GSH content of the supernatant measured using Eyer and Podhradsky's modification (1986) of the method of Tietze (1969). This involves reduction of the aromatic disulphide 5,5′-dithiobis(2-nitrobenzoic acid) (DTNB) to the chromogenic TNB by GSH, which is oxidised to its disulphide-linked dimeric form GSSG. In order to maintain the reaction, GSSG is reduced back to GSH by

Correspondence: D.W. Hedley, Dept of Medicine, Royal Marsden Hospital, Downs Road, Sutton, Surrey, UK.
Received 13 March 1989; and in revised form 30 June 1989.

addition of glutathione reductase and NADPH. After equilibration at room temperature the reaction was started by the addition of glutathione reductase, and the linear increase in absorbence at 412 nm seen using the test sample compared to that obtained using a standard containing reduced GSH. Results were expressed as fmol GSH per cell.

*Flow cytometry*

Monochlorobimane (mBCl, Molecular Probes, Eugene, OR, USA) was made up at 1 mM in 100% ethanol, and stored at −20°C. Cells were suspended at $1 \times 10^6$ $ml^{-1}$ in fresh tissue culture medium and stained using mBCl at various concentrations at 20°C for 2.5–30 min. Flow cytometry was done using a FACS Analyzer (Becton-Dickinson, Mountain View, CA, USA). This instrument uses a mercury arc lamp as the light source, and is capable of simultaneous measurement of cell fluorescence and Coulter volume. The strong 360–380 nm ultraviolet emission from the arc lamp was selected using a UG1 excitation filter, with a 480–500 bandpass filter being used for emission. Alignment was done using 9 µm diameter Hoechst-33342 stained calibration beads (Flow Cytometry Standards Corporation, Research Triangle Park, NC, USA), and fluorescence intensity expressed as channel numbers, which are arbitrary units on a linear scale. Immediately following the calibration beads, and using the same instrument settings, unstained cells were run to record autofluorescence, followed by the mBCl-stained cells, which were run in the presence of stain. A minimum of 30,000 stained cells was run, and mean cellular fluorescence (minus mean autofluorescence) recorded.

*Clinical samples*

A total of 14 clinical samples were examined. Seven of these were obtained by 23 gauge needle aspiration from patients who gave verbal informed consent for the procedure, one consisted of peripheral blood lymphoblasts separated by density-gradient centrifugation from a patient with end-stage T-cell acute lymphoblastic leukaemia, and the remainder were fresh surgical specimens which were mechanically disaggregated using crossed scalpel blades. Cell suspensions were filtered through gauze, washed and counted. If necessary, clumps were dispersed by syringing through 25 gauge needles. After adjusting to a final concentration of $1 \times 10^6$ $ml^{-1}$, cells were filtered through 70 µm plankton netting and stained using 40 µM mBCl for exactly 5 min at 20°C. Flow cytometry was done exactly as for cultured cells, except that the machine was triggered on Coulter volume, set at a threshold level of 300 $\mu m^3$ to exclude red blood cells and cellular debris. In addition to fluorescence, the ratio of cell fluorescence to Coulter volume was obtained using the electronics of the FACS Analyser.

### Results

*Monochlorobimane staining of EMT6 Cells*

The effects of mBCl concentration and time on the staining intensity of EMT6 cells are shown in Figure 1. There was a rapid initial increase in fluorescence, the rate slowing after approximately 15 min. Using 5 or 10 µM mBCl staining intensity then decayed, while with higher concentrations it slowly increased up to 30 min. Shrieve *et al.* (1988) have previously shown that when EMT6 cells are stained with 40 µM mBCl for 5 min, fluorescence intensity is closely correlated with biochemically determined GSH content, because the staining reaction is strongly catalysed by glutathione-S-transferase. We were able to confirm that >99% of cytoplasmic fluorescence was of low molecular weight by sonicating EMT6 cells stained with mBCl under the above conditions, fractionating with a Sephadex G25 column, and measuring fluorescence using a spectrofluorimeter. Furthermore, fluorescent material was eluted off the column in the same fractions

**Figure 1** Effects of time and concentration on mean fluorescence of EMT6 cells stained with monochlorobimane at 20°C. Indicated monochlorobimane concentrations are micromolar.

as a GSH-mBCl adduct pre-formed by reacting equimolar concentrations of the two compounds non-enzymatically. In contrast, when the more reactive GSH probe monobromobimane was substituted for mBCl, approximately 8% of cytoplasmic fluorescence was present in high molecular weight fractions.

*Biochemical assay of GSH*

The biochemical assay for GSH gave typical mean values of 3–4 fmol per EMT6 cell during exponential growth, falling to approximately 2 fmol $cell^{-1}$ when the cells were grown to high densities. This fall was not simply due to a reduction in cell volume, which had a mean value of approximately 3,900 $\mu m^3$ during logarithmic growth, and 3,500 $\mu m^3$ at high densities. Although treatment with 50 µM BSO for 6 h reduced the GSH content of exponentially growing cells by only approximately 50%, it was observed that similar treatment of the high density cells could yield mean GSH levels as low as 0.1 fmol $cell^{-1}$. The explanation for this apparently enhanced sensitivity to BSO at high density growth is unclear, but preliminary experiments suggest that it is not the direct result of medium exhaustion or acidification.

*Comparison of mBCl fluorescence and GSH assay*

The relationship between FCM-determined fluorescence of EMT6 cells stained with 40 µM mBCl for 5 min, and for cells from the same culture flask assayed for GSH is shown in Figure 2. Results for GSH contents of <3.0 fmol $cell^{-1}$ give a close linear fit intercepting almost at the origin (correlation coefficient = 0.969). Using this relationship, it can be seen that individual 9 µm calibration beads have a fluorescence equal to that of a GSH content of 0.83 fmol in an EMT6 cell stained with 40 µM mBCl for 5 min at 20°C. Note that for cell GSH contents >3.0 fmol $cell^{-1}$ (and possibly >2.0 fmol $cell^{-1}$) this linear relationship between fluorescence and GSH content does not hold. FCM tending to underestimate GSH content.

*FCM measurement of GSH in human cancer biopsies*

Of the patients studied, five had non-Hodgkin's lymphomas, four being diffuse large cell and one T-cell lymphoblastic lymphoma in terminal leukaemic phase. With the exception

**Figure 2** Correlation between mean fluorescence of mBCl-stained EMT6 cells and their glutathione content determined using a biochemical assay. The range in GSH content was obtained by depletion using BSO. Fluorescence is expressed as a ratio to that of 9 μm Hoechst-33342 stained calibration beads. The linear regression line excludes samples of GSH content greater than 3.0 fmol cell$^{-1}$.

of one patient with diffuse large cell lymphoma, all had recurrent disease following previous chemotherapy. There were nine solid tumours (two non-small lung, two cervix and one prostate, breast, endometrium, ovary and mesothelioma). Only two of these patients had received prior chemotherapy. Cells were stained using 40 μM mBCl for 5 min at 20°C. Because cell suspensions from human tumour biopsies contained large amounts of debris and red blood cells, the FACS Analyser was triggered on Coulter volume with a threshold set at 300 μm$^3$.

Measurement of individual cell fluorescence showed considerable heterogeneity. In contrast to EMT6 cells, where fluorescence intensity was roughly proportional to cell size, there was no obvious relation between Coulter volume and mBCl staining (Figure 3). Indeed, in many cases those cells

**Figure 3** Flow cytometric measurement of cellular glutathione content in a fine needle aspiration biopsy from a breast carcinoma. Top left indicates Coulter volume, threshold set at 300 μm$^3$ to exclude debris and red blood cells. Top right is glutathione content in arbitrary units on a linear scale. The correlated dot plot shows that the wide range in GSH content is not a simple function of cell size, the largest cells having relatively weak fluorescence. Subsequent cellular DNA content analysis showed that approximately 90% of cells were aneuploid, indicating that this heterogeneity did not result from contamination by normal host cells.

with the greatest volume showed only weak fluorescence, indicating that this wide range of values was not due to the presence of cell clumps. Restaining the sample illustrated in Figure 3 for cellular DNA content showed that approximately 90% of the cells were aneuploid, i.e. there was minimal contamination with normal host cells.

The relative fluorescence of the calibration beads was recorded immediately before running each of the clinical samples, and because these were stained under identical conditions to the EMT6 cells, the relationship between fluorescence and GSH content shown in Figure 2 was used to approximate mean GSH content of the tumour biopsies. This gave a mean value of 0.21 ± 0.12 fmol cell$^{-1}$ for the lymphomas, and 0.95 ± 0.39 for the solid tumours. Scatter of mean values is shown in Figure 4. Note that all samples were well within the linear part of the fluorescence/GSH content plot for EMT6 cells.

### Discussion

The ubiquitous presence of large amounts of GSH in cells reflects the need to detoxify free radicals generated by, for example, incomplete reduction of molecular oxygen or by certain xenobiotics. Because many anti-cancer agents mimic the actions of these natural threats to cells, it is not surprising that GSH and its related enzymes are implicated in clinical drug or radiation resistance. Interestingly, there is now evidence linking increased GSH-S-transferase or GSH peroxidase activities to enhanced *mdr* gene expression (Kramer *et al.*, 1988), and to capacity for DNA repair (Deffie *et al.*, 1988), suggesting that multifactorial cancer cell resistance could simply be the recruitment of a multi-layer defence system which evolved to protect macromolecules from common environmental hazards.

Despite an extensive literature examining the role of GSH in drug or radiation resistance in experimental model systems, surprisingly little has been published concerning fresh human cancer specimens. It would be important to determine the extent to which tumour cell GSH content caused clinical drug or radiation resistance, because methods for overcoming this have been proposed. In particular, GSH depletion using BSO can improve the therapeutic index of alkylating agents

**Figure 4** Estimated mean glutathione content of human cancer biopsies. Values were obtained by comparing mean cell fluorescence to that of 9 μm Hoechst-33342 stained calibration beads, and assuming that the linear relation between this and actual GSH content obtained for EMT6 cells holds for the clinical material.

used to treat human tumour xenografts, and phase 1 trials of BSO in cancer patients are underway.

Measurement of GSH using mBCl is an unusual FCM method, because its specificity depends on GSH-S-transferase combining it to GSH rather than to other sulphydryls. Recent evidence (Cook *et al.*, 1989) would suggest in fact that for at least some cell lines this, rather than GSH content, determines mBCl fluorescence. Cellular GSH turns over quite rapidly, and GSH feedback inhibits its own synthesis. Following reaction with an electrophile, GSH can be degraded, and its constituent amino acids recycled (Meister & Anderson, 1983). The complex time and concentration dependence of EMT6 cells illustrated in Figure 1 probably reflects these processes, since the act of staining cells with mBCl would be expected to cause depletion of target molecule. The higher concentrations of mBCl used exceed the total GSH content of the cell sample by several fold, and might therefore stimulate resynthesis as they deplete cells of existing GSH. Rice *et al.* (1986) showed that following its depletion by diethylmaleate, GSH content of EMT6 cells approximately doubled every 60 min. In contrast, the lower concentrations of mBCl might become exhausted as the GSH-mBCl adduct is degraded. It seems possible therefore that the assay is giving an overall picture of a cell's ability to cope with a reactive chemical species rather than simply measuring GSH. A more detailed picture of the processes involved might be obtained by the additional use of other sulphydryl stains such as mercury orange (O'Connor *et al.*, 1988), which, although probably less specific for GSH, stains non-enzymatically at 4°C. Despite the fact that an unusual dependence on a number of enzymes means that equilibrium between fluorochrome and target molecule is probably never achieved, EMT6 cells stained for 5 min using 40 $\mu$M mBCl had a mean fluorescence which correlated closely with biochemically determined GSH content over the range 0.2–2.0 fmol cell$^{-1}$. We have linked this correlation to the fluorescence of calibration beads, and subsequently used these beads as a tentative standard to quantitate clinical samples. The results show mean tumour cell GSH contents similar to those obtained using standard assays, with solid tumours having significantly higher values than non-Hodgkin's lymphomas. It should be emphasised that a large series comparing flow cytometry of human cancer biopsies (rather than EMT6 cells) with a standard GSH assay would be required in order to establish the reliability of quantitative FCM results. Nevertheless, we believe that the data shown here are of interest because they demonstrate a wide range in individual tumour cell GSH content which is not a simple function of cell size. The assay can be performed rapidly using fine needle aspirates, thus considerably extending its clinical scope by, for example, allowing sequential measurements to be made during treatment with agents designed to modulate cellular GSH. Under the latter circumstances exact quantitation of GSH may in fact be less critical, since results could be related to pre-treatment values. Although these results are preliminary, they suggest an emerging role for flow cytometry as an aid to active cancer patient management, in addition to its more defined place as an adjunct to diagnostic pathology.

### References

COOK, J.A., PASS, H.I., RUSSO, A., IYPE, B.A. & MITCHELL, J.B. (1989). Use of monochlorobimane for glutathione measurements in hamster and human tumour cell lines. *Int. J. Radiat. Oncol. Biol. Phys.*, **16,** 1321.

DEFFIE, A.M., ALAM, T., SENEVIRATNA, C. & 5 others (1988). Multifactorial resistance to adriamycin: relationship of DNA repair, glutathione transferase activity, drug efflux, and p-glycoprotein in cloned lines of adriamycin-sensitive and -resistant P338 leukaemia. *Cancer Res.*, **48,** 3595.

EYER, P. & PODHRADSKY, D. (1986). Evaluation of the micromethod for determination of glutathione using enzymatic cycling and Ellman's reagent. *Anal. Biochem.*, **153,** 57.

KRAMER, R.A., ZAKNER, J. & KIM, G. (1988). Role of the glutathione redox cycle in acquired and *de novo* multidrug resistance. *Science*, **241,** 694.

LEE, F.Y.F., ALLALUNIS-TURNER, M.J. & SIEMAN, D.W. (1987). Depletion of tumour *versus* normal tissue glutathione by buthionine sulphoximine. *Br. J. Cancer*, **56,** 33.

MEISTER, A. & ANDERSON, M.E. (1983). Glutathione. *Ann. Rev. Biochem.*, **52,** 711.

O'CONNOR, J.E., KIMLER, B.F., MORGAN, M.C. & TEMPAS, K.J. (1988). A flow cytometric method for intracellular nonprotein thiols using mercury orange. *Cytometry*, **9,** 529.

OZOLS, R.F., LOUIE, K.G., PLOWMAN, J. & 4 others (1987). Enhanced melphalan cytotoxicity in human ovarian cancer *in vitro* and in tumour-bearing nude mice by buthionine sulphoximine depletion of glutathione. *Biochem. Pharmacol.*, **36,** 147.

RICE, G.C., BUMP, E.A., SHRIEVE, D.C., LEE, W. & KOVACS, M. (1986). Quantitative analysis of cellular glutathione by flow cytometry utilising monochlorobimane: some applications to radiation and drug resistance *in vitro* and *in vivo*. *Cancer Res.*, **46,** 6105.

SHRIEVE, D.C., BUMP, E.A. & RICE, G.C. (1988). Heterogeneity of cellular glutathione among cells derived from a murine fibrosarcoma or a human renal cell carcinoma detected by flow cytometry. *J. Biol. Chem.*, **263,** 14107.

SKAPEK, S.X., COLVIN, O.M., GRIFFITH, O.W., ELION, G.B., BIGNER, D.B. & FRIEDMAN, H.S. (1988). Enhanced melphalan cytotoxicity following buthionine sulphoximine-mediated glutathione depletion in a human medulloblastoma xenograft in athymic mice. *Cancer Res.*, **48,** 2764.

TIETZE, F. (1969). Enzymatic method for quantitative determination of nanogram amounts of total and oxidised glutathione: applications to mammalian blood and other tissues. *Anal. Biochem.*, **27,** 502.